# New insights on collagen structural organization and spatial distribution around dental implants: a comparison between machined and laser-treated surfaces

**DOI:** 10.1186/s12967-024-04906-4

**Published:** 2024-01-31

**Authors:** Alessia Belloni, Giulio Argentieri, Giulia Orilisi, Valentina Notarstefano, Elisabetta Giorgini, Gianmaria D’Addazio, Giovanna Orsini, Sergio Caputi, Bruna Sinjari

**Affiliations:** 1 Department of Life and Environmental Sciences, Polytechnic University of Marche, Ancona, Italy; 2 Electron Microscopy Laboratory, Department of Innovative Technologies in Medicine and Dentistry, University “G. d’Annunzio” Chieti-Pescara, Chieti, Italy; 3 Department of Clinical Sciences and Stomatology, Polytechnic University of Marche, Ancona, Italy

**Keywords:** Dental implants, Collagen, Implant surface, Synthegra, Healing abutments, Fourier Transform InfraRed Imaging spectroscopy, Transmucosal surface

## Abstract

**Background:**

One of the main factors for the osseointegration of dental implants is the development of an adequate soft tissue barrier, mainly composed by collagen, which protects the implant from bacterial development. The structural features of the peri-implant collagen are influenced by the implant components and, in particular, by the type of the surface. In the clinical practice, healing abutments are characterized by smooth surfaces, named machined. Recently, a new laser technique, Synthegra, has been developed to obtain a topography-controlled surface with micrometric regular pores that seems reducing the risk of peri-implantitis. Based on this background, this study aims investigating the structural organization and spatial distribution of collagen surrounding healing abutments characterized by laser-treated and machined surfaces.

**Methods:**

Gingiva portions surrounding custom-made healing abutments (HA), characterized by alternated laser-treated and machined surfaces, were collected and analyzed by combining Fourier Transform InfraRed Imaging (FTIRI) spectroscopy, a non-invasive and high-resolution bidimensional analytical technique, with histological and multivariate analyses.

**Results:**

Masson’s trichrome staining, specific for collagen, highlighted a massive presence of collagen in all the analyzed samples, evidencing a surface-related spatial distribution. The nature of collagen, investigated by the FTIRI spectroscopy, appeared more abundant close to the laser-treated surface, with a perpendicular disposition of the bundles respect to the HA; conversely, a parallel distribution was observed around the machined surface. A different secondary structure was also found, with a higher amount of triple helices and a lower quantity of random coils in collagen close to the laser treated surfaces.

**Conclusions:**

FTIRI spectroscopy demonstrates that the use of a laser treated transmucosal surface can improve the morphological organization of the peri-implant collagen, which presents a distribution more similar to that of natural teeth.

*Trial registration*: This trial is registered with ClinicalTrials.gov Identifier: (Registration Number: NCT05754970). Registered 06/03/2023, retrospectively registered, https://clinicaltrials.gov/show/NCT05754970.

## Background

Modern literature agrees that implantology is a highly reliable and predictable procedure, with a survival rate of about 90% in a medium to long-term follow-up period [1]. However, dental implants are not completely free from biological and mechanical complications [2]. Biological failure has been defined as the inadequacy of the host tissue to establish or maintain osseointegration [3]. It can be classified into early and late failure. Early failure can occur in the first few weeks or maximum a few months after implant placement. It may depend on the intrinsic properties of the host tissue; in general, it is caused by a biological agent which hinders surgical wound healing and, hence, prevents the achievement of the osseointegration [4]. Late failure refers to an implant that has lost the achieved osseointegration due to the onset of peri-implantitis [5], an inflammatory pathological condition that affects the peri-implant soft and hard tissues leading to the progressive loss of supporting bone and thus to implant failure [6]. Peri-implant mucositis develops from healthy peri-implant mucosa as a result of bacterial biofilm accumulation around osseointegrated dental implants [7]. Peri-implant disease shows an etiopathogenetic mechanism and its risk factors are very similar to periodontal disease [8–10]. Studies in dogs have shown that the inflammatory infiltrate in peri-implant tissues is greater than that which develops in periodontal tissues [5, 11, 12]. The inflammation at the level of the implants arises because there are no supracrestal fibers to stem the disease as in periodontitis. In addition, unlike periodontitis, in peri-implantitis inflammation also occurs at the bone level (osteitis), again due to the lack of these supracrestal fibers, which act as a barrier [5].

Collagen stands as the predominant fibrous protein in the extracellular matrix in all mammalian connective tissues; it forms an essential framework used by fibroblasts as scaffolding to "crawl" along [13, 14]. It serves as the primary structural component, crucial for upholding tissue mechanical properties, offering tensile strength, and overseeing vital processes like cell adhesion and tissue development [15]. Structurally, collagen presents a triple-stranded helical arrangement, comprising three left-handed α-chains coiled around each other to form a right-handed super-helix [16, 17].

As collagen plays a pivotal role in the structure and function of the ECM, any irregularities in its composition or organization inevitably lead to altered tissue characteristics. One of the main differences between the gingival tissues surrounding a natural tooth and a dental implant is the collagen fiber orientation around the collar of the device just below the gingival surface. In a natural tooth, dentogingival collagen fibers are inserted into the cementum and the bone, and oriented perpendicular or oblique to the tooth surface [18, 19]. In contrast, around a dental implant they are mainly parallel to the implant surface [20, 21].

The adhesion of the soft tissue to the implant, called transmucosal attachment, acts as a seal which prevents damages to osseointegration processes, avoiding the formation and attachment of microorganisms to the bone tissue [22]. It represents the result of a surgical wound healing, unlike the soft tissues around the tooth which develops at the same time as the periodontium [23, 24]. Many studies have shown how the structural features of the implant, such as the surface chemistry and topography, the macro- and micro-designs of abutments [25], the wettability [26] and the presence of biologically active proteins [27–30], can influence the adhesion of the junction epithelium and of the connective tissue to the abutment surface [31].

The literature agrees that surface roughness can influence cell adhesion [32]. Among all, laser treatment, a procedure which causes an increase of the surface roughness, has been shown to be very beneficial both for reducing bacterial contamination [33] and maintaining the mechanical characteristics [34]. In this light, the Synthegra technique, a recently developed procedure based on a new generation of laser technology, has shown promising results [35, 36]. The laser, moving along the implant surface, removes titanium by sublimation and creates thousands of micrometric pores with regular distribution and equal in shape and size: micropores have a diameter of 5 microns, an interpore distance of 15 microns and a depth of 5 microns. The result is a clean, topography-controlled surface that induces excellent osseointegration and significantly reduction of bacterial adhesion, resulting in a lower risk of peri-implantitis [36].

Fourier Transform Infrared Imaging (FTIRI) spectroscopy is a well assessed and label-free tool for studying non-homogeneous biological samples such as tissues and cells [37, 38]. In fact, the coupling between infrared spectroscopy and visible microscopy and the development of array detectors able to reach a spatial resolution down to a few microns, let obtain high-resolution IR images which simultaneously provide reliable information on the biomolecular composition and structural organization of several macromolecules, including proteins and collagen [17, 39]. Recently, FTIRI has been successfully applied for exploring the structural organization of collagen in soft and hard tissues and interesting results have been obtained [37, 38].

In this light, in the present study, we propose a multidisciplinary approach which combines FTIRI spectroscopy with histological and multivariate analyses to evaluate ex vivo the effects of healing abutments with conventional and experimental surfaces (i.e. machined and laser-treated) on the structural and macromolecular organization of collagen. The null hypothesis of this study is that the collagen surrounding the laser-treated surface of the healing abutment (HA) exhibits qualitative and quantitative characteristics, in terms of organization, distribution, and orientation, comparable to those observed in collagen in contact with the machined surfaces.

## Methods

### Patients’ selection

N. 7 patients were selected among the thirty who participated to the first step [36]. Inclusion criteria were as follows: patients aged between 18 to 75 years; good oral and systemic health; at least 2 mm of adherent gingiva in the site chosen for rehabilitation; adequate residual bone ridge. Conversely, exclusion criteria were the following: patients with scarce oral hygiene; Full Mouth Plaque Score and Full Mouth Bleeding score greater than 25%; active periodontal disease; uncontrolled diabetes mellitus; number of cigarettes smoked per day greater than 10.

All patients, enrolled by the Implantology Operating Unit of the Department of Medical, Oral and Biotechnological Sciences, “G. d’Annunzio” University of Chieti-Pescara, signed a written informed consent to participate. The study, conducted according to the principles established in the Declaration of Helsinki (2008) developed by the World Medical Association on Humans, was approved by the Ethics Committee of the University of Chieti-Pescara “G. d’Annunzio” (18/10/2018, No. 22).

### Surgical treatment

Before surgery, patients were submitted to oral physical and radiographic examinations, such as Orthopantomography and Cone Beam Computed Tomography. The implant placement surgery was performed by an experienced surgeon (GDA) under local anesthesia obtained by infiltration of a 4% articaine solution containing 1:100,000 adrenaline. Through the preparation of a full-thickness flap, exposure of the bone ridge was achieved. The implant site was prepared by helical drills with increasing diameters to the desired measure under copious irrigation. All inserted implants (Omny, Geass srl, Pozzuoli del Friuli, Udine, Italy), had different diameters (3.50 mm, 4.1 mm) and lengths (7.0 mm, 8.0 mm, 10.0 mm, 11.5 mm). After screwing the plug screws onto the implants these were submerged. Patients were given oral hygiene instructions; antibiotic therapy (amoxicillin + clavulanic acid 2 g/day) and rinses with mouthwash with 0.20% chlorhexidine (Chlorexidine^®^, Oral-B, Boston, MA, USA) were prescribed for seven days. The appointment for suture removal was scheduled at 7–10 days after surgery.

**Fig. 1:**
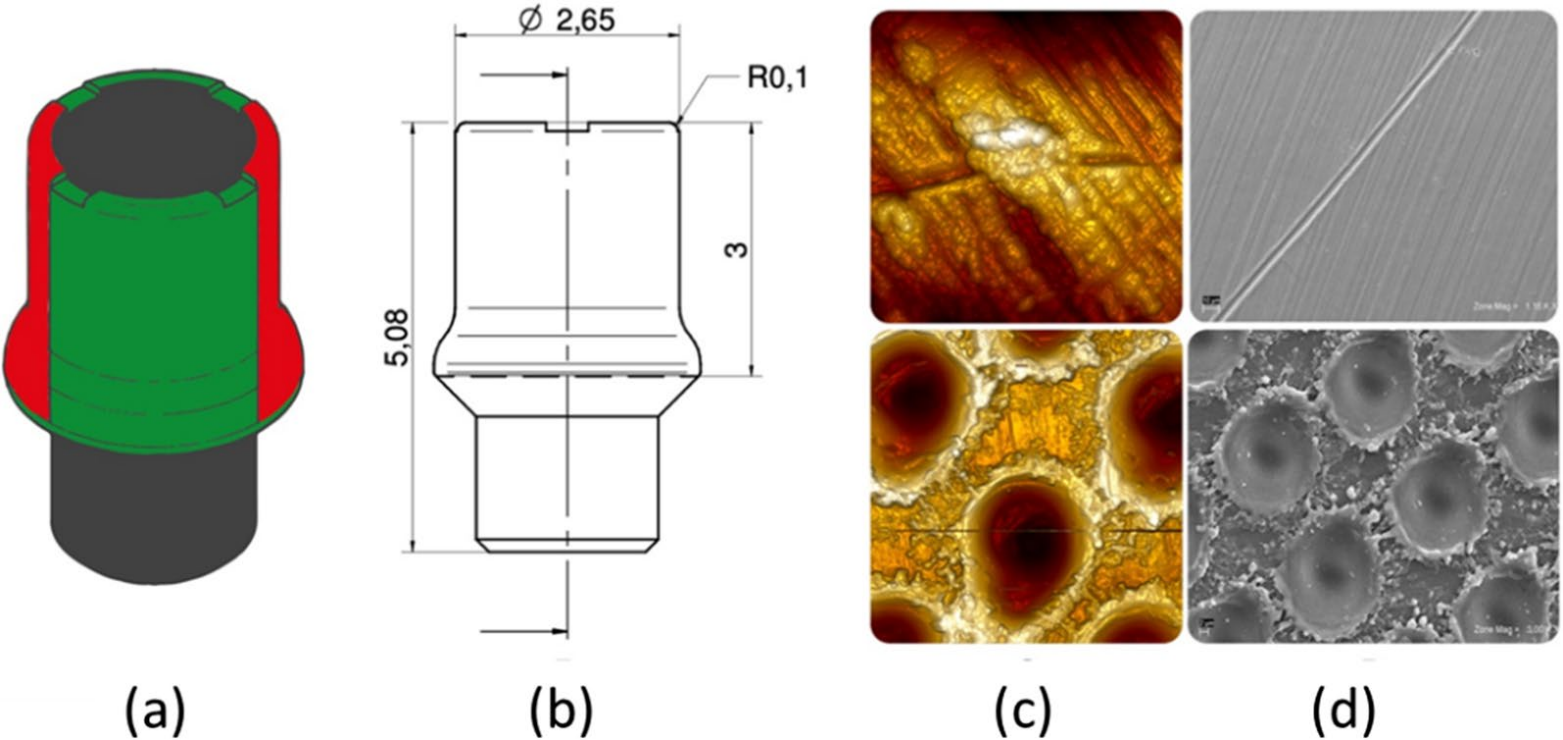
**a** Schematic description of the experimental healing abutment surface treatments (laser-treated in red and machined in green). **b** HA technical design and dimensions. **c** Atomic force microscope images of the two surface treatments (up-machined and down laser-treated). **d** Scanning electron microscope images of the machined (up) and laser-treated (down) surfaces used in the experimental HA

The second surgical phase was performed after a healing period of 12 ± 4 weeks. After uncovering the implants, the cap screws were replaced by experimental HAs, made of Grade 5 titanium (TiVaAl alloy) and with a diameter of 2.65 mm and a height of 5.08 mm (Geass srl, Pozzuoli del Friuli, Udine, Italy). The surface of these experimental HAs was subjected to two different treatments, to obtain on each HA two laser-treated (Synthegra technique) and two machined surfaces, alternating with each other (Fig. 1).

### Samples collection and preparation

**Fig. 2:**
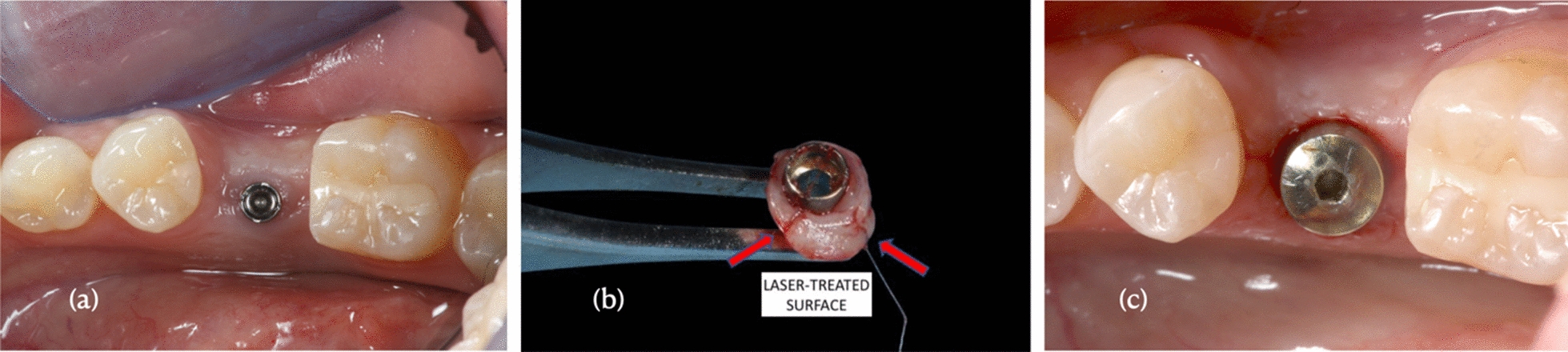
**a** Experimental HA (diameter of 2.65 mm and height of 5.08. mm). **b** Image of a representative post-operative portion of gingiva surrounding the dental implant with alternating machined and laser-treated surfaces, as indicated by the arrows. **c** The experimental healing abutments were replaced by a standard healing HA, taking care to preserve the keratinization tissue obtained during healing

After the healing period (30 ± 7 days) [40], HAs were retrieved together with the corresponding gingival biopsies collected by a 5-mm diameter circular punch. In order to have a correct orientation of the biopsies, two 7/0 sutures (OMNIA S.r.l. S. Michele Campagna Fidenza, Parma, Italy) were used to delineate the portion of gingiva in contact with one of the two laser-treated surfaces (Fig. 2).

**Fig. 3:**
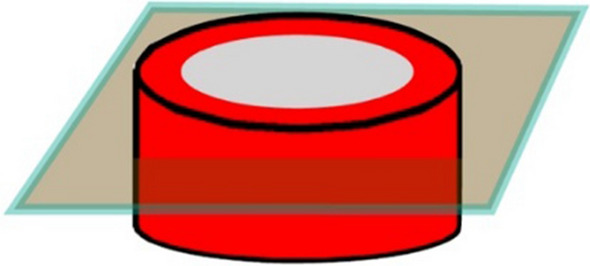
Sketch representing a paraffin block of gingival tissue (red coloured) and the transverse plane (grey square) along which the cut was made with the microtome to obtain 7 μm-thickness sections

Gingiva portions were fixed by immersion in 4% paraformaldehyde solution at 4 °C for 24 h. Then, they were washed once with 0.9% NaCl (w/w) for 5 min, twice with 70% ethanol (w/w) for 10 min, and finally stored in 70% ethanol (w/w). After dehydration with ethanol gradient [17], samples were washed with xylene (Bio-Optica, Milan, Italy), and finally detached from the healing screw and embedded in paraffin (Bio-Optica, Milan, Italy), paying attention to preserve the correct orientation and to memorize the position of the laser-treated and machined surfaces. The obtained paraffin blocks were cut according to the transverse plane (see Fig. 3) by using a microtome (Leica RM2125RTS, GmbH, Wetzlar, Germany); from each block, six 7 μm-thickness sections containing all the four alternated surfaces were obtained, and alternatively deposited onto glass slides and CaF_2_ optical windows (13 mm diameter, 1 mm thickness), respectively for histological and FTIRI analyses.

### Histological analysis

Seven-µm thickness sections were stained with Masson’s trichrome staining (Bio-Optica, Milan, Italy), specific for collagen which results blue coloured. The stained sections were examined by means of a Zeiss Axio Imager.A2 microscope (Zeiss, Oberkochen, Germany). The images were acquired by using the Axiocam 503 combined color digital camera (Zeiss, Oberkochen, Germany).

### FTIRI measurements

FTIRI measurements were carried out at the Laboratory of Vibrational Spectroscopy of the Department of Life and Environmental Sciences, Polytechnic University of Marche (Ancona, Italy). A Bruker INVENIO-R interferometer coupled with a Hyperion 3000 Vis-IR microscope and equipped with a Focal Plane Array detector for tissue imaging analysis, operating at liquid nitrogen temperature (Bruker Optics, Ettlingen, Germany), was used.

The photomicrograph of each section was acquired with a 15 × objective; photomicrographs were used to accurately select specific areas, corresponding to the gingival tissue close to the machined (named MACHINED) and laser-treated (named LASER) surfaces, on which acquiring IR images. The acquisition was performed in transmission mode in the 4000–800 cm^–1^ spectral range (spectral resolution 4 cm^–1^); each IR image was 164 × 164 μm and consisted of 4096 pixels/spectra, with a spatial resolution for each pixel of 2.56 × 2.56 μm; each pixel/spectrum was the result of 256 scans. Before proceeding with the analysis of the sample, the background spectrum was acquired on a clean region of the CaF_2_ optical window, using the same setup (OPUS 7.5 software package, Bruker Optics, Ettlingen, Germany). IR images were opportunely processed to avoid both the signals due to the presence of atmospheric carbon dioxide and water vapor, and artifacts caused by even slight differences in section thickness (respectively Atmospheric Compensation and Vector Normalization routines; OPUS 7.5 software package, Bruker Optics, Ettlingen, Germany).

False-color images were generated by integrating pre-processed IR images under the 1300–1185 cm^–1^ region, to evaluate the spatial distribution of collagen within the mapped areas. Pre-processed IR images were also subjected to Hierarchical Cluster Analysis (HCA), using Euclidean distance and Ward's method (CytoSpec software v. 2.00.01). By comparing IR images with the relative histological ones, the clusters corresponding to collagen-rich area were identified, and the corresponding IR spectra extracted. For each patient, the mean IR spectra of each laser-treated and machined surface were calculated, together with their standard deviation spectra (Averaging routine, OPUS 7.5 software package, Bruker Optics, Ettlingen, Germany).

Mean spectra were the vector normalized and curve fitted in the 1730–1590 cm^–1^ and 1360–1180 cm^–1^ spectral ranges, to highlight changes in collagen secondary structure. The number and position of the underlying peaks were defined by the analysis of second derivative spectra and fixed during the curve fitting procedure; a Gaussian distribution was adopted for peak shape (GRAMS/AI 9.1, Galactic Industries, Inc., Salem, New Hampshire). Peaks’ assignment was performed in accordance with data reported in the literature [41–44]. The areas (A) of the most significant peaks were calculated and used to obtain specific spectral parameters (see “Results” section).

### Statistical analysis

The sample size calculation was performed according to Covani et.al 2023 [45]: a sample size of 14 statistical units (surface treatment) per group was calculated to have a minimum difference of collagen fiber quantity ($\mu m$ ^2^) between the two groups. The α value was determinate as 0.05 while the power of the test was 0.95. For the statistical calculation Gpower sample size software calculation was used.

Statistical analysis was performed using PRISM 6.0 software (GraphPad Software, San Diego, CA, USA). All data were presented as mean ± SD. Statistical significance between groups was assessed using the t-test. Statistical significance was set at p < 0.05 (*p < 0.05; **p < 0.01; ***p < 0.001 and ****p < 0.0001; ^n.s.^p > 0.05).

## Results

**Fig. 4:**
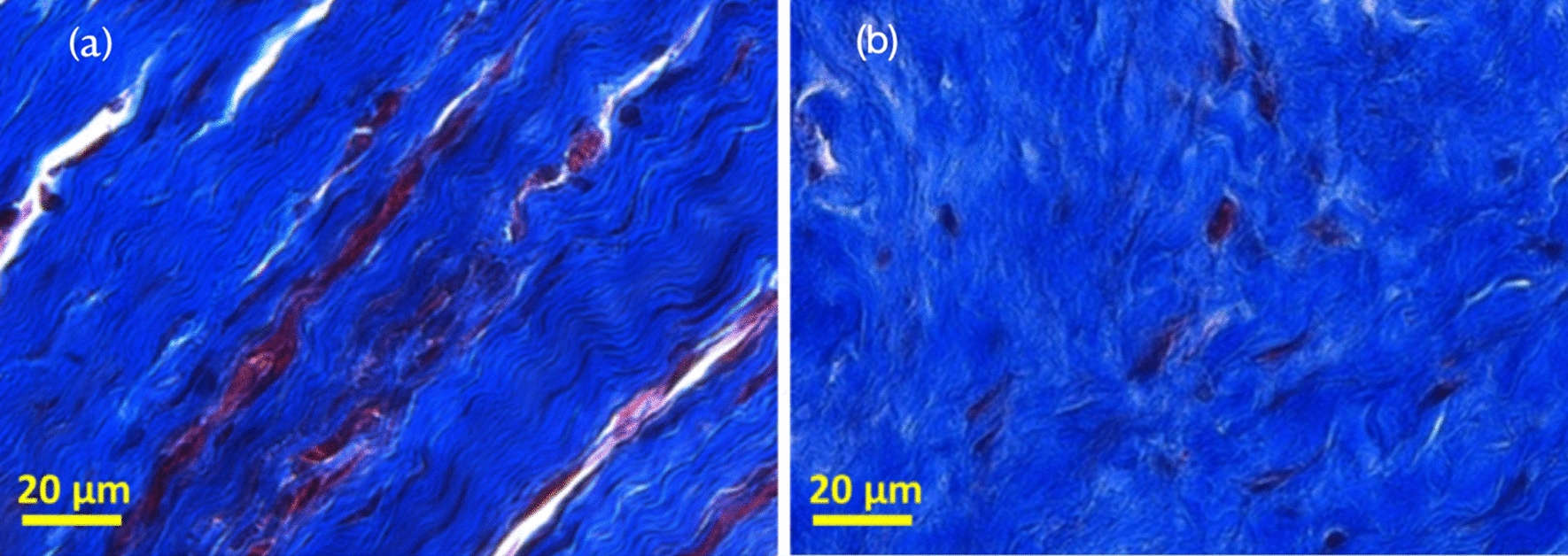
Masson’s trichrome histological images of representative sections of gingival tissue adjacent to (**a**) machined and (**b**) laser-treated implant surfaces (20 × magnification)

Representative Masson’s trichrome histological images of gingival tissue adjacent to dental implants with alternated laser-treated and machined surfaces, are displayed in Fig. 3. In all the sections, a massive presence of collagen was observed, even if with a distinctive arrangement and distribution in relation with the two different implant surfaces. More in detail, a well evident longitudinal disposition is displayed by collagen bundles close to the machined surface (Fig. 4a); conversely, no specific orientation was found in collagen in the presence of the laser-treated surface, even if some bundles seem to be perpendicular to the HA (Fig. 4b).

**Fig. 5:**
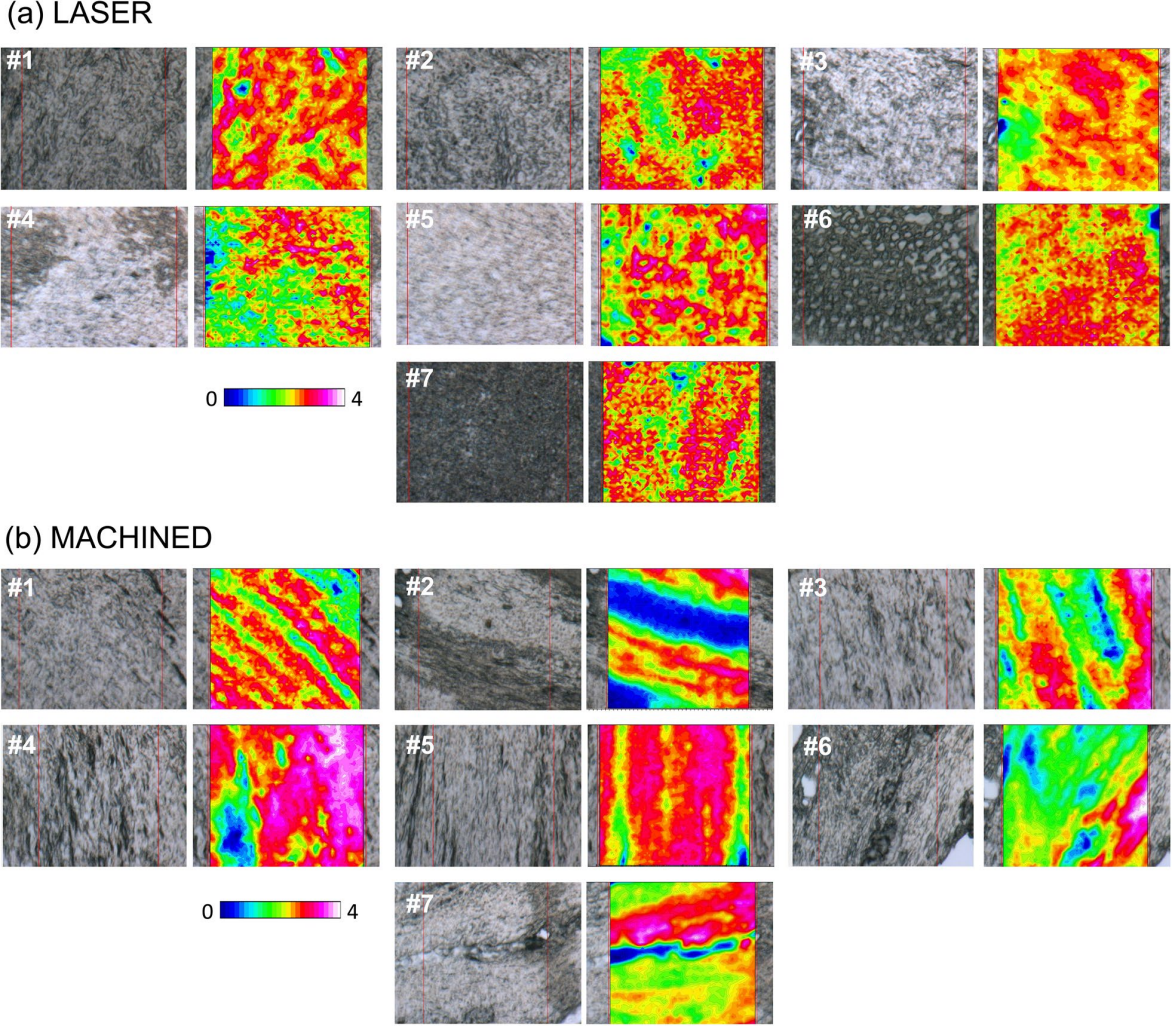
Hyperspectral analysis of the IR images collected on gingival portions adjacent to (**a**) laser-treated (LASER) and (**b**) machined (MACHINED) surfaces. For each patient (#1–#7), the photomicrographs of representative LASER and MACHINED areas are reported (on the left), together with the false color images showing the topographical distribution of collagen (on the right). The red squares in the photomicrographs indicate the areas on which IR images were collected (164 × 164 μm^2^; 4096 pixel/spectra; spatial resolution 2.56 × 2.56 μm^2^). An arbitrary colored scale was adopted, white/pink colors indicating areas with the highest amount of collagen, while blue/black colors those with the lowest one

More information on the composition and spatial distribution of collagen bundles was obtained by the hyperspectral analysis of the IR images. In Fig. 5, the photomicrographs of representative LASER and MACHINED areas for each patient (#1–#7), are reported, together with the false color images showing the spatial arrangement of the collagen bundles. False color images are a useful tool to evidence the topographical distribution of specific macromolecules, including proteins, collagen, lipids, and so on. They are generated by integrating the whole IR image under defined spectral intervals, specific for each macromolecule. The arbitrary color scale also allows to visualize the relative quantity of the investigated macromolecule, with white/pink indicating areas with higher levels and blue/black areas with lower ones. In this study, the IR images were integrated under the 1300–1185 cm^–1^ region, which includes the most diagnostic vibrational modes of collagen. Their analysis confirmed that collagen is the main component of gingival tissue, and that collagen bundles show a perpendicular orientation in the tissue adjacent to the laser-treated surface (Fig. 5b), while in the machined surface they exhibit a distribution longitudinal to the implant (Fig. 5b).

**Fig. 6:**
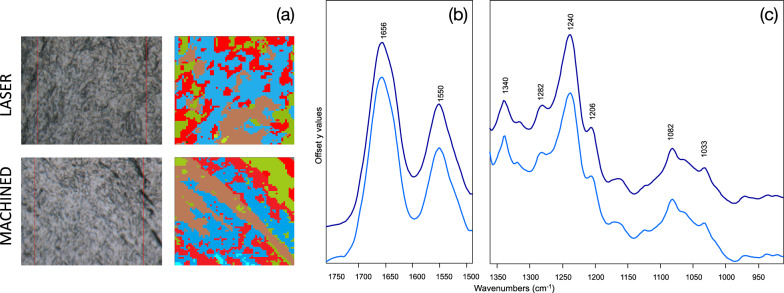
**a** Hierarchical Cluster Analysis of IR images representative of LASER and MACHINED samples (light blue color indicates the clusters richer in collagen). Mean absorbance IR spectra extracted from the clusters richer in collagen of LASER (light bleu lines) and MACHINED (blue lines) samples: **b** 1760–1490 cm^–1^ and **c** 1360–900 cm^–1^ spectral ranges. Spectra are offset along the y axis for a better comprehension. The position of the most significant bands is indicated over the spectra (wavenumbers, cm^−1^)

IR images were also submitted to Hierarchical Cluster Analysis (HCA), to accurately identify the areas with the highest amount of collagen, from which to extract the IR spectra. In Fig. 6a, as an example, the HCA of representative LASER and MACHINED samples is shown, different colors indicating the presence of four clusters, each characterized by a peculiar spectral profile: the clusters richer in collagen were light blue colored.

The mean absorbance IR spectra extracted from the clusters richer in collagen corresponding to LASER and MACHINED samples were analyzed in the most significant spectral ranges: 1760–1490 cm^–1^ representative of total proteins (Fig. 6b), and 1360–900 cm^–1^ representative of collagen (Fig. 6c). The bands centred at ~ 1656 cm^–1^ and ~ 1550 cm^–1^, named respectively Amide I and II bands are related to the vibrational modes of the functional groups involved in the peptide bond (i.e. C=O, C–N and N–H) (Fig. 6b). The band at ~ 1340 cm^–1^ is attributable to the CH_2_ groups in proline, the most abundant amino acid in collagen; also the bands at ~ 1282 cm^–1^, ~ 1240 cm^–1^ and ~ 1206 cm^–1^, named Amide III, with a typical tricuspid shape, are mainly attributed to collagen (Fig. 6c). Finally, the bands at ~ 1082 cm^–1^ and ~ 1033 cm^–1^ are related respectively to phosphate groups in phosphorylated compounds and C–OH bonds in carbohydrates (Fig. 6c) [17].

**Fig. 7:**
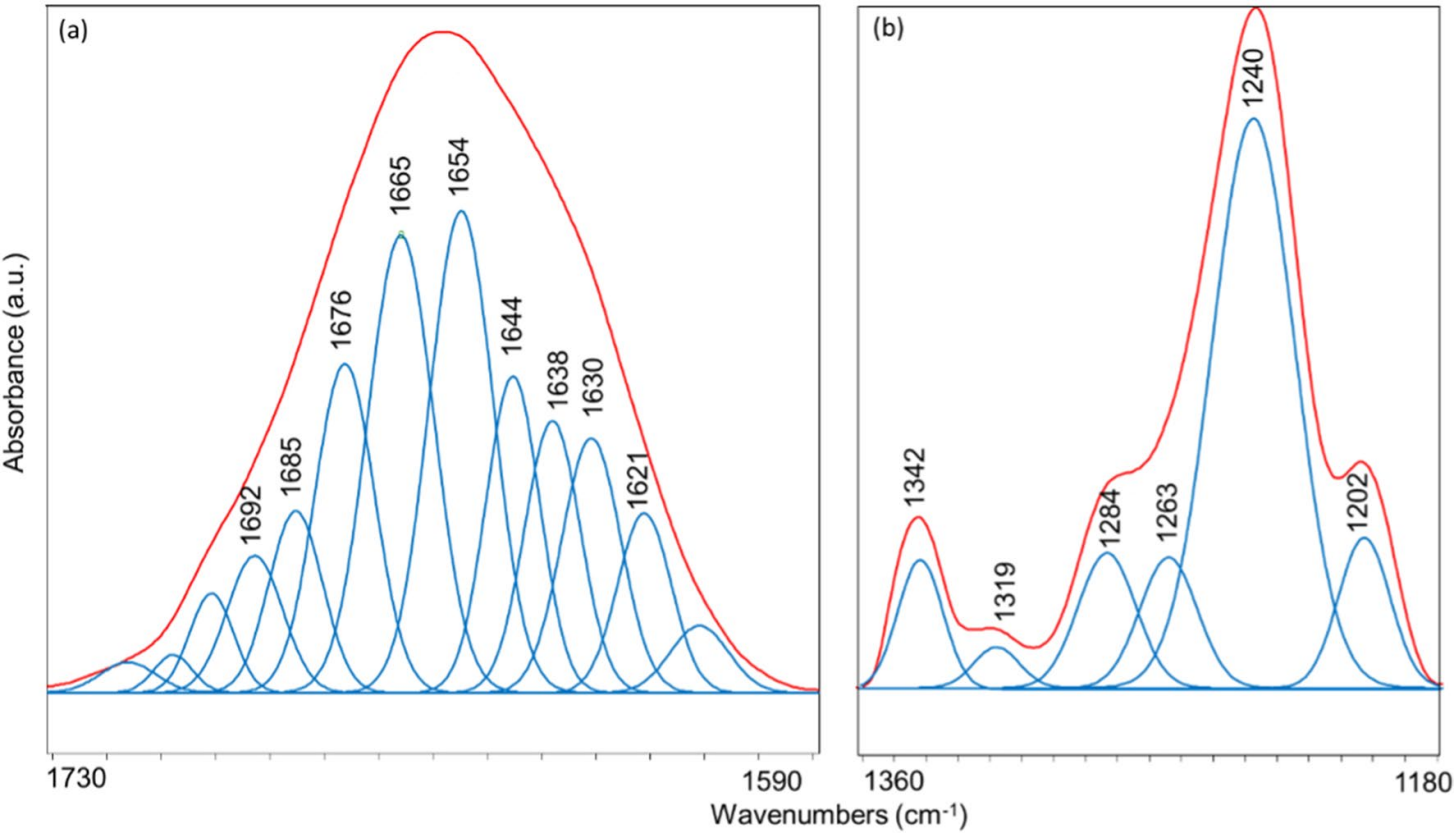
An example of curve fitting analysis performed on mean IR spectra of LASER and MACHINED samples in the (**a**) 1730–1590 cm^−1^ and (**b**) 1360–1180 cm^–1^ spectral ranges (the red line is the original spectrum, while blue lines indicate the subcomponent peaks, whose position is reported over peaks as wavenumbers)

Mean spectra were then curve fitted in the following spectral ranges: 1730–1590 cm^–1^ and 1360–1180 cm^–1^ (Fig. 7). Curve fitting is a well assessed procedure which let identify the subcomponent peaks underlying a convoluted band. The first spectral interval (1730–1590 cm^–1^) corresponds to the Amide I band and provides relevant and reliable information on proteins secondary structure [17, 46–48]. In particular, the bands at ~ 1692 cm^–1^, ~ 1685 cm^–1^, ~ 1630 cm^–1^ and ~ 1621 cm^–1^ are assigned to β-sheet structures, while those at ~ 1665 cm^–1^ and ~ 1654 cm^–1^ to α helix ones; moreover, the bands at ~ 1676 cm^–1^ and ~ 1638 cm^–1^ are attributable respectively to 3-turn helix and triple helix structures; finally, the band at ~ 1644 cm^–1^ is due to random coil structures. As regards the 1360–1180 cm^–1^ range, it includes the Amide III band and is representative of the structural features of collagen [17, 49, 50]. In this spectral region, the following bands were detected: ~ 1342 cm^–1^ (wagging of CH_2_ groups in proline side chains); ~ 1319 cm^–1^ (α helix structures of collagen); ~ 1284 cm^–1^ and ~ 1240 cm^–1^ (triple helix structures of collagen); ~ 1263 cm^–1^ (random coil structures), and ~ 1202 cm^–1^ (lateral chains of aminoacids) [17].

Based on curve fitting data, the following spectral parameters were calculated: A_1665+1654_/A_AI_ (ratio between the sum of the areas of the bands at 1665 cm^–1^ and 1654 cm^–1^ and the area of the 1730–1590 cm^−1^ range, corresponding to the Amide I band and named AI); A_1644_/A_AI_ (ratio between the area of the band at 1644 cm^–1^ and the area of the Amide I band, calculated as described above); A_1638_/A_AI_ (ratio between the area of the band at 1638 cm^–1^ and the area of the Amide I band, calculated as described above); A_1342_/A_tot_ (ratio between the area of the band at 1342 cm^–1^ and the area of the 1360–1180 cm^–1^ range, named A_tot_); A_1284+1240_/A_tot_ (calculated as ratio between the sum of the areas of the bands at 1284 cm^–1^ and 1240 cm^–1^ and A_tot_, calculated as described above), and A_1263_/A_tot_ (ratio between the area of the band at 1263 cm^–1^ and A_tot_, calculated as described above).

**Fig. 8:**
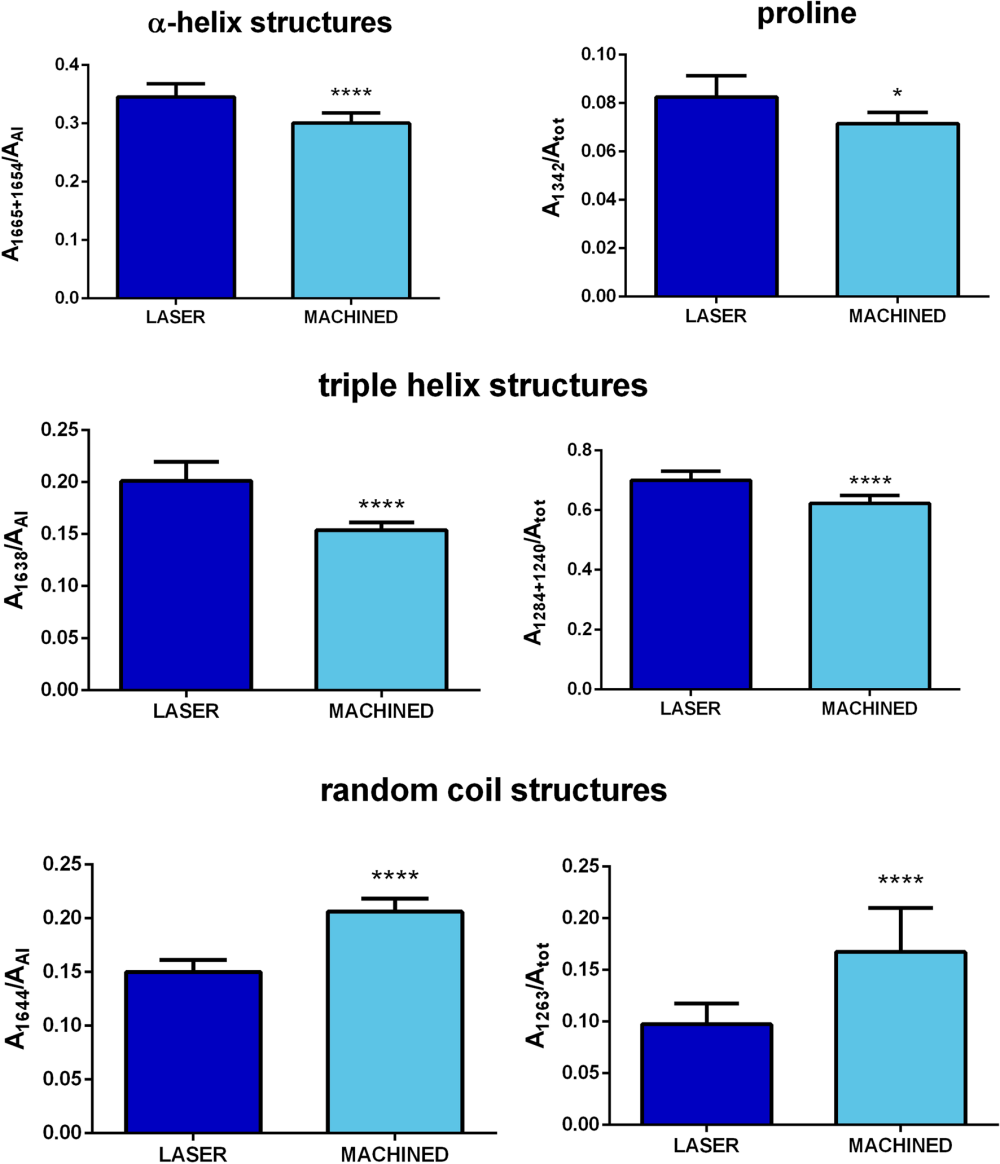
Statistical analysis (t-test) of proteins and collagen secondary structure in gingival portions adjacent to laser-treated (LASER) and machined (MACHINED) surfaces: α-helix structures (A_1665+1654_/A_AI_), proline (A_1342_/A_tot_), triple helix structures (A_1638_/A_AI_ and A_1284+1240_/A_tot_), and random coil structures (A_1644_/A_AI_ and A_1263_/A_tot_). Data are shown as mean ± SD; asterisks over histograms indicate statistically significant differences (*p < 0.05; **p < 0.01; ***p < 0.001 and ****p < 0.0001)

The statistical analysis of these spectral parameters is reported in Fig. 8. The ratios A_1665+1654_/A_AI_ and A_1342_/A_tot_ indicating, respectively the relative amount of α-helix structures and proline, can be associated with the relative amount of collagen. Both show higher values in the LASER samples respect to MACHINED ones, confirming a greater quantity of collagen in the tissue near the laser-treated surface (A_1665+1654_/A_AI_, p < 0.0001; A_1342_/A_tot_, p < 0.05). The ratios A_1638_/A_AI_ and A_1284+1240_/A_tot_ are representative of the relative amount of triple helices, respectively in proteins and collagen, and hence they can provide information on the structural organization of collagen. Both ratios display higher values in LASER samples respect to MACHINED ones, letting hypothesize the presence of a more organized collagen in the tissue close to the laser-treated surface (A_1638_/A_AI_, p < 0.0001; A_1284+1240_/A_tot_, p < 0.0001). Finally, the ratios A_1644_/A_AI_ and A_1263_/A_tot_ are attributable to random coil structures, respectively in proteins and collagen. For both ratios, lower values were detected in LASER samples respect to MACHINED ones (A_1644_/A_AI_, p < 0.0001; A_1263_/A_tot_, p < 0.0001), suggesting the presence of unfolded protein structures in the latter. Hence, the higher number of alpha and triple helices, and the lower one of random coil structures observed in LASER samples respect to MACHINED ones, let hypothesize that the laser-treated surface could promote the formation of a greater amount of collagen, with a more organized structure.

## Discussion

Over the years, new configurations and topographic characterizations have been proposed and investigated to improve the soft tissue attachment to the transmucosal part of dental implants [51–53]. A satisfactory bone-to-implant connection could not alone guarantee a long-term success of dental implants [3, 54]. Indeed, the clinical success is also related to the quality of soft tissues surrounding the implant neck and to the orientation of the peri-implant collagen fibers [55]. After implant insertion, gingival fibroblasts begin to proliferate, repopulate, and generate a collagen-rich extracellular matrix in the transmucosal region that adheres to the HA [56, 57]. This physical barrier between the oral cavity and the osseous support of the implant, is represented by collagen fibers, whose well-organization were hypothesized to decrease early bone resorption by reducing bacteria infiltration, due to the formation of a soft tissue barrier [5, 6, 58]. For this reason, the surface characteristics of the HA play an important role in a strategic area of deep tissue remodeling, by creating a biologic width and influencing collagen fiber orientation [59, 60]. Until now, in implantology, the scientific literature has not clarified whether it is preferable to have a transgingival implant portion with controlled roughness or a machined one [61]. According to some authors, the transgingival portion with a machined surface may reduce the adhesion of bacteria and the consequent risk of bacterial colonization and inflammation of soft and hard tissues around dental implant [62, 63]. From this perspective, a machined implant collar could reduce the occurrence of peri-implantitis [64]. At this purpose, in this study, the machined surface of HA was considered as control and compared with the laser-treated, considered the experimental surface [65].

The results of the present study support the rejection of the null hypothesis. Indeed, in all the analyzed cross-sections, the histological analysis evidenced a massive presence of collagen in the tissue around the HA, with a surface-related spatial pattern: a well evident longitudinal disposition is displayed by collagen bundles close to the machined surface, while in the presence of the laser-treated surface, collagen does not show a specific orientation, even the majority of the bundles seem to be perpendicular to the HA. These differences were better highlighted by the analysis of the IR images, which confirmed that collagen is the main component of all the analyzed samples, and that bundles exhibited a longitudinal distribution to the implant in the machined surface, whereas in the tissue adjacent to the laser-treated one, the fibers are randomly distributed, with a prevalence of perpendicular orientation. It is well known that while moderate crosslinking positively impacts collagen fiber mechanical properties, excessive crosslinking renders these fibers more fragile [66]. Moreover, several authors have suggested in human and animal histologic studies that collagen circular orientation around HA could not stabilize the peri-implant connective tissues [67, 68]. Indeed, the fibers aligned in a parallel orientation around dental implants could be considered not functional, whereas the functional physical orientation is represented by a perpendicular pattern [69, 70]. This kind of attachment is similar to that of a natural tooth, which is an indispensable barrier against bacterial infection and other harmful stimuli.

It is, also, known that transmucosal connective tissue integration is achieved by a small number of gingival fibroblasts (only 3–5%) in mature peri-implant connective tissue, which is significantly lower than the concentration of gingival fibroblasts in healthy periodontal connective tissue (approximately 15%) [71]. The lack of fiber connection to the abutment surface and the lower amount of gingival fibroblasts produce a poorly integrated transmucosal connective tissue layer on the dental implant abutments. The enhancement of fibroblast functions can be reached through appropriate topographical/biological modifications of the abutment faces [32, 72]. Our previously human clinical studies evaluated the biological advantages of using transmucosal components with a rough surface characterized by a controlled topography formed by micrometric regular pores. This kind of surface, obtained with the Synthegra technique, have demonstrated a positive influence on cell adhesion [36] and a reduced inflammatory infiltrate [35], suggesting a good chance of positively influencing the difficult coronal biological seal formation. The present results are in line with the previously published one and enhance the importance of surface treatment on the biological structural organization of the peri-implant soft tissues.

In this study, a higher amount of collagen was found in the laser-treated surface (A_1342_/A_tot_ and A_1665+1654_/A_AI_ band area ratios, relative, respectively to proline and α-helices). Evidence of a different secondary structure was also highlighted by the univariate analysis of specific band area ratios. In particular, collagen close to the laser-treated surface is characterized by the presence of a major number of triple helices (A_1638_/A_AI_ band area ratio) and less random coil structures (A_1644_/A_AI_, p < 0.01 band area ratio), letting hypothesize that this collagen is more similar to the native one [42].

A limitation of the current study may be the short follow-up (12 ± 4 weeks) which does not allow to compare the intensity of the cellular events in peri-implant tissues after implant loading. However, it is reported that the peri-implant soft tissue clinical maturity is established 4 weeks following implant placement by a one-stage surgical protocol [73].

Thus, our findings, supported by previous literature, suggest that during extracellular matrix maturation around the HA, the local microenvironment could be influenced by the different HA surface treatments and that the collagen orientation near the laser-treated surfaces could allow a better preservation of the peri-implant bone resorption.

However, further studies are necessary to better understand and characterize the gingival tissues around HA, focusing on the peri-implant connective fibers orientations.

## Conclusions

The results of this study demonstrate how the use of a laser-treated transmucosal surface can improve the morphological organization of peri-implant soft tissue. In fact, the collagen tissue formed around the surface of the laser-treated abutment, in addition to being present in greater quantity, also displayed a more organized distribution with a higher amount of triple helices, respect to the collagen near the machined one. This finding let hypothesize a good degree of maturation, similar to that found in natural teeth.

Even if all these findings suggest that the laser-treated surface can promote the formation of a soft tissue able to provide a better seal, further studies will be needed to fully understand the role of such a topography-controlled surface for improving this sealing and increasing the performance and longevity of dental implants.

## Data Availability

The datasets used and/or analyzed during the current study are available after request to the corresponding author.

## Abbreviations

HA: Healing abutments
FTIRI: Fourier Transform InfraRed Imaging
HCA: Hierarchical cluster analysis
